# Importance of Acute Anterior Angulation in Double Aortic Arch Needing Attention at Primary Surgery

**DOI:** 10.3389/fcvm.2021.760523

**Published:** 2022-01-24

**Authors:** Arun Beeman, Madhavan Ramaswamy, Yadav Srinivasan, Siddartha Rudrappa, Georgi Christov, Jan Marek, Graham Derrick, Nagarajan Muthialu

**Affiliations:** Department of Cardiothoracic Surgery, Great Ormond Street Hospital for Children, London, United Kingdom

**Keywords:** vascular ring, double aortic arch, tracheooesophageal symptoms, division of double aortic arch, tracheal compression

## Abstract

**Background:**

Vascular rings are rare congenital abnormalities of the aortic arch. There are many embryological variants including a double aortic arch. In symptomatic children, division of ring and release of airway structures may be sufficient. Persistence of symptoms can be related to an anterior angle formed between the two arches. The aim of this study is to evaluate the clinical efficacy in improving symptoms and on changing this angle at the primary surgery.

**Methods:**

All children who had surgery for double aortic arch between 2005 and 2020, were studied. Relevant factors were analyzed for persistent symptoms including anatomical substrates and surgical details.

**Results:**

A total of 87 out of 224 children had surgery for a double aortic arch. At presentation, airway symptoms (*n* = 74/87) were more common than esophageal symptoms (*n* = 27/87). Early onset symptoms within 1 year were seen in 49 children. In addition to division of one arch, surgical steps also included realigning the anterior left arch, thereby eliminating the acute angle in 36 children (after 2014). After surgery, symptom relief within 12 months following surgery was seen in 64% of children (56 out of 87) but in 27 out of 36 children (75%) with additional surgical modification, as against 29 out of 51 (57%) in those with division of the arch. Symptoms persisted beyond 1 year needing reintervention in eight children.

**Conclusion:**

Anterior arch angulation plays an important role in double aortic arch by causing a “nutcracker” phenomenon. Repair in double aortic arch should consider this aspect and include modification of surgical steps by realigning the corresponding aortic arch branches and an anterior pexy in selected cases.

## Introduction

Vascular rings are rare congenital abnormalities of aortic arch and its branches. By circumferentially compressing the trachea and esophagus, they cause a wide range of obstructive aero-digestive symptoms. Although this compression can be relieved by standard surgical division of the ring, the symptoms may continue to persist in some children long after the repair. This has a huge impact on the quality of life of the children and their caregivers.

Various factors are responsible for the symptoms to continue after surgery. Among them the most important reason is pre-operative tracheobronchomalacia. This is expected to improve over time, although in some it can persist for many years. Other anatomical factors that play a role in persistence of symptoms include circumflex morphology of the right aortic arch, persistence of Kommerell's diverticulum (which was not operated on at the time of the first surgery) and scarring after the initial repair. One of the important anatomical substrates in double aortic arch is the acute anterior angulation between the right and left aortic arches.

Based on the persistence of symptoms from an earlier error, we incorporated the surgical idea of changing this geometry between the two arches anteriorly, and analyzed the results based on this changed practice.

## Materials and Methods

All children who had repair of double aortic arch as part of vascular ring surgery in our tertiary pediatric institution between 2005 and 2020 were included in the study. While true vascular ring includes double aortic arch (DAA) and right aortic arch with left retroesophageal ductal ligament (RAA) or any retro-esophageal vascular components, we used this total number for denominator and excluded those without anatomical variants where there are no complete two rings with or without patency. Compression of airway and esophagus caused by innominate artery, left pulmonary artery sling and aberrant right subclavian artery were also excluded from the study as they do not form a complete ring. Relevant information was collected retrospectively from case notes of the children. An institutional review board permitted exemption from the need for consent and ethical clearance in view of this being a retrospective study.

The factors included for the study were demographics, presenting symptoms, age at presentation, diagnostic investigations, associated abnormalities, morphology of the ring, age at surgery, surgical details, reintervention, and symptoms at follow-up. Additional anatomical factors analyzed include dominance and patency of the arches, and the angle subtended anteriorly between the right and left aortic arch. In addition to the above factors, the position and the course of descending thoracic aorta in relation to the trachea was analyzed. Although arbitrary, a 1-year period was chosen to be a reasonable time beyond which the symptoms if present were considered to be persistent. The data was analyzed by chi-squared test for significance of association between the risk factors and persistent symptoms.

### Surgical Technique

All children underwent surgery through thoracotomy. Left thoracotomy (85 out of 87) was commonly used as the approach, based on either right dominant anatomy or co-dominant circulation with left sided arterial duct. Two children underwent right thoracotomy due to anatomical left dominant aortic arch.

**Figure 1:**
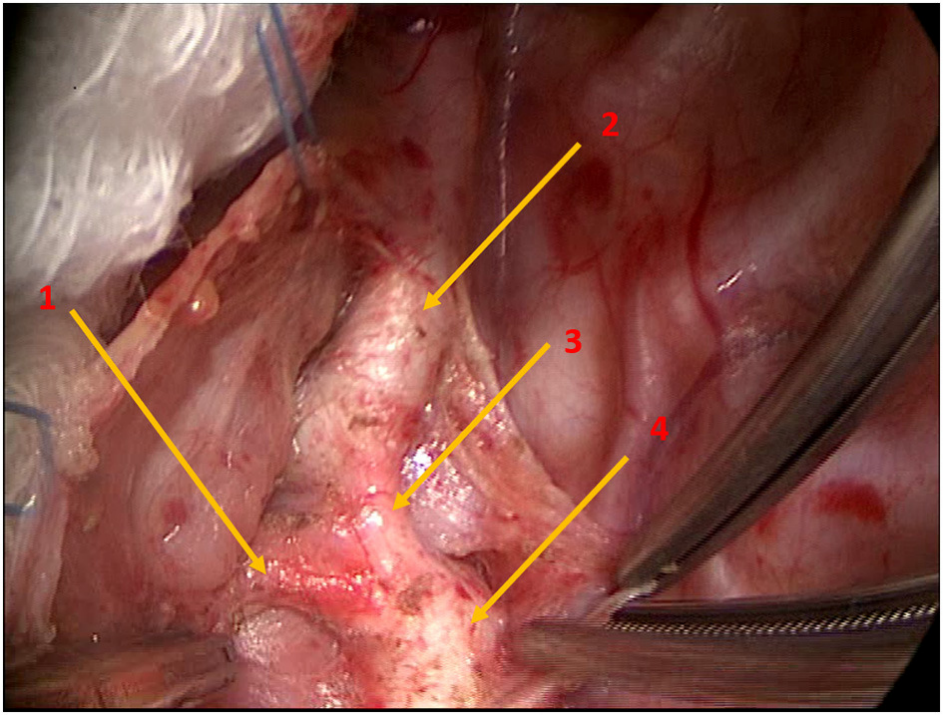
Intraoperative photograph demonstrating the anatomy of the left arch at surgery, including the transverse lie of the proximal left arch along with the origin of the left subclavian artery. Arrow 1: Ductal ligament; Arrow 2: Left subclavian artery; Arrow 3: Left aortic arch; Arrow 4: Descending thoracic aorta.

At surgery, the mediastinal pleura was divided and stayed. Gentle dissection allowed for clear description of anatomy and arterial duct was divided between ligatures. Following this, the distal left arch (or right arch in those two children) was divided between clamps from the position just after the subclavian artery and to the level of its connection to the posterior right aortic arch (Figure 1). After this, further gentle dissection to allow for release of the anterior left aortic arch was undertaken, thereby the atretic or non-dominant arch is fully released from the trachea-esophageal complex. Care is taken particularly to avoid/protect the recurrent laryngeal nerve. The distal most point at the level where the subclavian artery arises is used as the point of fulcrum, and pledgetted polypropylene suture was used to lift this to the anterior chest wall, thereby widening the acute angle formed by this anterior arch. The suture need not be very tight and the distal pulsatility of the subclavian artery is used as the guide for avoiding potential kink, so as not to allow this maneuver in causing any obstruction to flow.

The conventional double aortic arch surgery where the arch and duct were divided was done until 2014, when the new modification was introduced. Since 2014, all children who underwent surgery for double aortic arch included this important modification as part of the surgery.

## Results

Out of 224 children who underwent vascular ring surgery in the study period (2005 to 2020), 87 were operated on for double aortic arch. Table 1 shows basic demographic details of these children who had double aortic morphology. At presentation, airway symptoms (*n* = 74/87) were more common than esophageal symptoms (*n* = 27/87). Symptoms usually began in the first year of life in 49 out of 87 children.

**Table 1:** Baseline characteristics.

**Characteristics**	**Children with double aortic arch *n* = 87**
Median age (months)	8
Median weight at surgery (kilogram)	8.2
Female gender	37/87 (42.5%)
Onset of symptoms before 1 year	59/87 (67.8%)
Airway symptoms	74/87 (85.1%)
Esophageal symptoms	27/87 (31.0%)
Associated cardiac abnormalities	21/87 (24.1%)
Associated extra cardiac abnormalities	17/87 (19.5%)

The anatomical confirmation of the morphology was done by both preoperative echocardiography and computerized tomographic assessment of vascular anatomy. Evaluation included additional anatomical variables, as shown in Table 2. This includes patency of arches including their dominance and position of descending aorta.

**Table 2:** Baseline anatomical factors within double aortic arch morphology.

**Characteristics**	**Number out of total children with double aortic arch *n* = 87 (100%)**
Both arch patent	44 (50.6%)
Atretic segment	43 (49.4%)
Dominance of right arch	76 (87.3%)
Dominance of left arch	2 (2.3%)
Co-dominance of arches	9 (10.3%)
Left descending aorta	54 (62.1%)
Midline descending aorta	17 (19.5%)
Right descending aorta	16 (18.4%)

At surgery, in addition to division of one arch, surgical steps also included realigning the anterior left arch at the level of the left subclavian artery, thereby eliminating the acute angle, in 36 out of 87 children (after 2014).

After surgery, symptom relief within 12 months following surgery was seen in 56 out of 87 children (64%) overall. Between the error, 22 out of 51 children (43.1%) operated on until 2014 and only nine out of 36 (25%) operated on after 2014 with the modified technique remained symptomatic beyond 1 year from the operation (with a *p* value of 0.08 between these two groups).

Table 3 shows the details of recurrent symptoms, including the incidence of airway related vs. esophageal symptoms in these children. Additionally, the details of further surgical intervention are highlighted.

**Table 3:** Recurrence of symptoms and reintervention.

**Characteristics**	**Recurrent symptoms (*n* = 31)**
Airway symptoms	29/31
Esophageal symptoms	4/31
Left position of descending aorta	14 out of 31 with acute angulation
Reintervention	Total: 8 Anterior aortopexy: 5 Redo thoracotomy for scar/persistent fibrous strand: 3

Further surgical intervention included both the anterior approach for aortopexy and redo thoracotomy. This depended on the additional anatomical findings on repeat imaging. In this group of children who needed further surgical attention, three needed redo thoracotomies for attempts at releasing scars and five needed aortopexies from the anterior approach for moving the anterior aortic position away from the airway. Two had tracheostomies in view of persistent need for ventilator support for respiratory management. With the numbers being low, there was no further statistical calculation on this group of children needing reintervention.

One important anatomical substrate identified later during assessment was the presence of acute angulation at the arch, between right and left arches, in the context of the descending thoracic aorta being on the left side. While the significance of this is unclear, we presume this could potentially create a circumflex arch, after division, around the airway/esophagus, further precipitating symptoms.

Calculation of the anterior angle was undertaken retrospectively. This was done using the pre-operative and postoperative CT scans of the chest. Measurement of angles is not in a standardized way, though, due to various reasons, including availability and quality of images on the scan and alignment of two arches in the same plane in order to get an effective measurement of the angle.

### Statistics

Being a retrospective study, there are no powered calculations. We used mean and median for demographics with Chi-square plotting for calculating the statistical *p* value for significance between the two groups on outcome.

## Discussion

Management of a vascular ring has evolved since it was first repaired in 1945 by Dr. Robert Gross in Boston Children's Hospital (1). Despite advanced diagnostics and improved surgical techniques, a considerable number of children still continue to have symptoms after surgery (2–4).

Vascular ring morphology can be related to many variants of aortic arch development. Double aortic arch morphology is one of the most common in this broad spectrum of aortic arch disorders, which can present with symptoms very early in life (5–7). Persistence of an additional arch from fetal life can lead to a narrow space for trachea-esophagus complex, thereby leading to airway and/or esophageal symptoms (7, 8).

Pre-operative malacia is an important cause for the persistence of symptoms after surgery (9–11). This is expected to improve over time on its own, although in some, the airway symptoms linger on for many months. They can be worsened by inter-current infections and at times may require ventilator support. Aortopexy can alleviate the obstructive airway symptoms caused by airway malacia in a selected few (12, 13).

None of our patients needed any direct tracheal or bronchial intervention, specifically stenting of airways for persistent malacia. This is because once the inciting factor—aortic arch anomaly from double aortic arch entity—has been corrected, malacia can be expected to improve.

Other important causes for persist symptoms include scarring from previous surgery, Kommerell's diverticulum which was not repaired in the initial surgery, and a circumflex morphology which was not operated appropriately (2, 13). These can be addressed by relevant surgical techniques.

**Figure 2:**
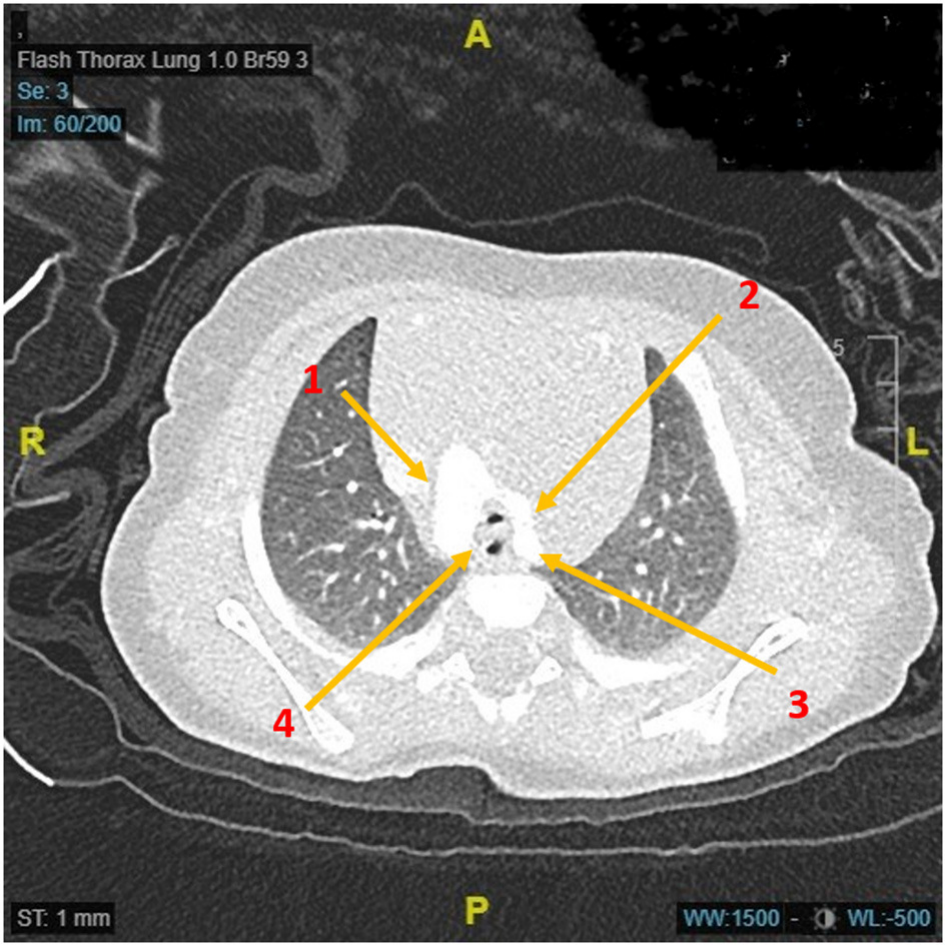
Axial computerized tomographic image demonstrating the double aortic arch and a very small tracheal lumen due to acute angulation between two anterior arches. Arrow 1: Right aortic arch; Arrow 2: Left aortic arch; Arrow 3: Origin of left subclavian artery; Arrow 4: Narrow trachea and esophagus in the vascular ring complex indicating narrow anterior angle.

Beyond the reasons above, there could still be other factors that are responsible for persistence of symptoms. One of the important morphological observations in double aortic arch, is the presence of acute angulation at the juncture of arches anteriorly. The acute angle formed between the right and left arches anteriorly, where the trachea is generally caught at, can potentially act like a “nutcracker” phenomenon causing pulsatile compression. Surgery, as is often performed through the left thoracotomy, addresses the distal left arch. This would leave the anterior remnant of the left arch unattended to, essentially leaving the angle intact (Figure 2). Hence symptoms often persist in DAA presumably, especially in smaller children, or where this angle can be quite acute.

Addressing this angle is of paramount importance, if this is relevant. In our series, out of 87 children who underwent surgery, 41 of them had persistent acute angle after surgery. In terms of error, 29 out of 51, who had surgery pre-2014 when the particular surgical modification was undertaken, had persistent angle. They went on to remain symptomatic beyond one year following surgery, needing further reintervention (three needing a redo thoracotomy for attempts at releasing the scar; five needed aortopexy from the anterior approach for moving the anterior aortic position away from the airway). Two had tracheostomy in view of the persistent need for ventilator support for respiratory management.

Since 2014, 36 children had undergone surgery for double aortic arch, where 27 of them were asymptomatic at the time of assessment (of 1 year). There were no reinterventions in the remaining nine children, and symptoms varied between mild and moderate respiratory symptoms including stridor but none of them were on any respiratory support.

Though attributable to chance association in such a short series of retrospective nature, this is a potential area of addressing a crucial anatomical problem, in what could be a long-lasting symptomatic disorder in a child. The technique would involve modification, whereby the non-dominant arch had to be moved away from its plane of orientation effectively releasing the nutcracker compression. By addition of this technique, the surgical time is not unduly prolonged, nor the procedure by itself is technically demanding to pose additional risk.

There would be a need for further longitudinal analysis in these children to ensure such a pathological assessment is valuable, and an additional surgical step can help in these struggling children following vascular ring surgery.

## Conclusion

Surgery for double aortic arch has been standardized with low operative morbidity and mortality. But symptom relief may not always be achieved well after surgery. Anterior arch angulation plays an important role in double aortic arch by causing a “nutcracker” phenomenon. Morphological variations can be a potential reason for persistence of symptoms and appropriate surgical attention helps in these children. Repair in double aortic arch should consider this aspect of anatomy and include modification of surgical steps by realigning the corresponding aortic arch branches and by including an anterior pexy in selected cases.

## Limitations

This is a retrospective case note review from a single center and a majority of these cases were operated by a single surgery in a major part of the study period. Data could be incomplete, though follow up data is complete. The study limits itself to the specified morphology and used only airway symptoms predominantly though esophageal symptoms were included wherever applicable. Measurement of angle is not a standardized calculation and further work needs to be done on this for better understanding of the impact of such morphological characteristics on clinical effect.

## Data Availability Statement

The raw data supporting the conclusions of this article will be made available by the authors, without undue reservation.

## Ethics Statement

The studies involving human participants were reviewed and approved by Institutional Ethics Committee (R&D) Great Ormond Street Hospital. Written informed consent to participate in this study was provided by the participants' legal guardian/next of kin.

## Author Contributions

AB, MR, and YS prepared the clinical data. YS and GC worked on data and helped with writing initial manuscript. JM and GD edited. NM conceptualized the initial concept, was the operating surgeon and supervised the entire manuscript work including editing the final version and submission. All authors contributed to the article and approved the submitted version.

## Conflict of Interest

The authors declare that the research was conducted in the absence of any commercial or financial relationships that could be construed as a potential conflict of interest.

## Publisher's Note

All claims expressed in this article are solely those of the authors and do not necessarily represent those of their affiliated organizations, or those of the publisher, the editors and the reviewers. Any product that may be evaluated in this article, or claim that may be made by its manufacturer, is not guaranteed or endorsed by the publisher.
